# Monitoring the genetic variation of some *Escherichia coli strains* in wild birds and cattle

**DOI:** 10.4102/ojvr.v90i1.2085

**Published:** 2023-07-26

**Authors:** Ghada A. Ibrahim, Ahmed M. Salah-Eldein, Mayasar I. Al-zaban, Amal S.A. El-Oksh, Elsayyad M. Ahmed, Doaa S. Farid, Enas M. Saad

**Affiliations:** 1 Bacteriology Department, Agriculture Research Center (ARC), Animal Health Research Institute, Ismailia, Egypt; 2 Wildlife and Zoo Department, Faculty of Veterinary Medicine, Suez Canal University, Ismailia, Egypt; 3 Department of Biology, College of Science, Princess Nourah bint Abdulrahman University, Riyadh, Saudi Arabia; 4 Biotechnology Department, Reference Lab of Quality Control of Poultry Production (RLQP), Agriculture Research Center (ARC), Animal Health Research Institute, Sharkia, Egypt; 5 Department of Virology, Animal Health, Research Institute (AHRI), Agricultural Research Center (ARC), Giza, Egypt; 6 Department of Environmental Protection, Faculty of Environmental Agricultural Sciences, Arish University, El-Arish, Egypt

**Keywords:** *E. coli*, wild birds, cattle, virulence genes, resistant genes, PCR, antibiotics, sequencing

## Abstract

To date, there is limited data about the genetic relationship of *Escherichia coli* between wild birds and cattle because these birds act as silent vectors for many zoonotic bacteria. This study aimed to elucidate the role of rooming wild birds in the vicinity of cattle farm in transmission of the same pathogenic *E. coli* variants, identifying their virulence, resistance traits and genetic similarities of *fim*H virulence gene. About 240 faecal/cloacal swabs were collected from both species and examined bacteriologically. *Escherichia coli* was yielded in 45.8% and 32.5%, respectively, of examined cattle and wild birds. The most prevalent detected *E. coli* serovar was O26. High tetracycline and chloramphenicol resistance were recorded; however, gentamycin and ciprofloxacin exhibited the highest sensitivity rates. Polymerase chain reaction (PCR) conserved genotypic resistance (*tet*A and *bla*_CTX_-M) and virulence attributes (*fim*H, *stx*1, *eae*A and *omp*A) of *E. coli* isolates were discussed in detail. The *fim*H gene revealed 100% sequence similarity when comparing with different *E. coli* isolates globally and locally. Finally, a close genetic association of *E. coli* with both wild birds and cattle was detected, thus strengthening its role in the dissemination of the infection via environment. Prevention and conservative policy should be carried as *E. coli* constitute enormous significant zoonotic risks to livestock and animal workers. Also, further studies to the whole genome sequencing of *fim*H, other virulence and resistance genes of *E. coli* are recommended trying to limit the possibilities of co-infection and transfer among different species.

**Contribution:**

The current study recorded updated data about the critical infectious role of wild birds to livestock, including cattle farms in Egypt. It also delivered some recommendations for good hygienic practices in cattle farms which must be implemented for handling animal manure.

## Introduction

Wildlife constitutes a magnificent network that might spread a number of potentially significant bacterial diseases with their antimicrobial-resistance traits among livestock animals and infrastructure because of high bird travel and mobilisation in multiple countries during different seasons (Arnold et al. 2016). Migratory and non-migratory wild birds could serve as the main transmitter for the infection with the family *Enterobacteriaceae* specifically *Escherichia coli* spp. (Shobrak & Abo-Amer 2014).

*Escherichia coli* is a Gram-negative environmental bacterium that commensally inhabits the intestinal tract of warm-blooded animals and birds. However, these bacteria could cohabit with other bacteria forming the commensal gut microbiota; some *E. coli* variants might develop virulent characteristics producing the disease (Umpiérrez et al. 2021).

Hence, the antimicrobial resistance problems could extremely extend to transfer the antibiotic-resistant genes among different species via plasmid or transposon (Borges et al. 2017). For antimicrobial resistance in the veterinary field, *E. coli* resistance was consistently the highest (Fahim et al. 2019). Furthermore, *E. coli* had also been regarded as a reliable indicator bacteria for tracking antibiotic resistance in domestic and wild animals. However, enteropathogenic (EPEC) and Shiga toxigenic *E. coli* (STEC) are categorised under diarrheagenic *E. coli*, and the intestinal microbiota of wild birds could include both serovars with their multi-drug resistance attributes (Borges et al. 2017). Moreover, the precise genetic bacterial profile enables more knowledge for the most disseminating genotypes to distinguish its potential links to virulence factors that could infect many animals and free-living birds (Skarżyńska et al. 2021).

As wildlife could play the main role in the microbial spreading in cattle farms, the mechanisms of this bacterial spreading among species in the farm environment are poorly understood (Tormoehlen et al. 2019). The current investigation was conducted to estimate the likelihood of *E. coli* infections transfer by wild birds that were frequently in contact with cattle and identifying the interrelationship between both species in farms in Egypt. Also, to study the most virulence and antibiotic resistance traits of the *E. coli* spp. isolates with a special regard to partial-genome resistance of *fim*H gene.

## Research methods and design

### Sampling

A total of 240 rectal and cloacal swab samples were collected from cattle species (*n* = 60 apparently healthy, *n* = 60 diarrhoeic cattle) and 120 from different resident wild birds from Ismailia Governorate, Egypt. Cows and buffaloes were included in this study of native breeds. Resident free-living wild birds (Hooded crow [*n* = 21], cattle egret [*n* = 18], spur-winged plover [*n* = 24], pied kingfisher [*n* = 21], green bee-eater [*n* = 24] and stone curlew [*n* = 12]) were captured with nets nearby these cattle farms and released after swabbing. Animals were housed in open yards surrounded by a fence, partially covered with sheds and muddy floors. All cattle rectal and bird cloacal swabs were collected aseptically and transferred immediately under hygienic measures to the laboratories of the Animal Health Research Institute (AHRI) Ismailia branch in ice thermal boxes for bacteriological examination, serological and polymerase chain reaction (PCR) identification.

### Bacterial cultivation and identification

All cattle rectal and bird cloacal swabs were enriched overnight onto buffered peptone broth and then incubated at 37 °C under aerobic conditions. Subsequently, the cultures were isolated (on MacConkey, EMB and sheep blood agar plates) and identified using conventional techniques (Quinn et al. 2002). Furthermore, the pathogenicity of each one of *E. coli* recovered isolates was evaluated separately onto Congo red agar plate medium then they were kept overnight at 37 °C. The cultures were kept at room temperature for 48 h (Singh et al. 2014). After 48 h at room temperature, Congo red-positive pathogenic *E. coli* isolates appeared with a brick red colour while non-pathogenic ones were colourless (Saha et al. 2020).

### Serological identification

The recovered *E. coli* isolates were serotyped based on their ‘O’ antigen according to the manual of the Reference Lab for Veterinary Quality Control on Poultry Production, Animal Health Research Institute, Dokki, Egypt. The identified isolates were preserved in tryptone broth 1% with adding glycerol to a final concentration of 15%. The tubes were kept at −20 °C for further analysis.

### Antimicrobial susceptibility testing

The purified *E. coli* isolates were characterised for antibiotic susceptibility on Mueller–Hinton agar plates by the disc diffusion method using 11 different antibiotic discs (tetracycline, chloramphenicol, amoxiclavulinic acid, penicillin, piperacillin, streptomycin, fosfomycin, gentamycin, danofloxacin ciprofloxacin and cefepime), which were selected according to the panel of antibiotics of interest to the dairy industry and public health in our country. The results were interpreted using the standard guidelines of the Clinical and Laboratory Standards Institute (CLSI 2020).

### Polymerase chain reaction screening of virulence and resistant traits of *Escherichia coli* spp.

Following the manufacturer’s instructions of QIAamp DNA Mini Kit (QIAGEN, Germany), the genomic deoxyribonucleic acid (DNA) of *E. coli* bacterial isolates was extracted. The PCR was performed using thermal profiles and oligonucleotides primers (Table 1) and a 25 μL reaction volume containing; 12.5 μL Master Mix (EmeraldAmp Max PCR, Takara, Japan), 1 μL (20 pmol) from each forward and reverse primer (Metabion, Germany), 5.5 μL Dnase free water and 5 μL DNA template. The amplified genes fragments were visualised in ethidium bromide-stained agarose gel 1.5% against 100 base pair (bp) DNA ladder (GeneDirex, Taiwan) in 1× Tris-borate EDTA (TBE) buffer at 100 voltage (V) per 30 min and then recorded using the SynGene Gel Documentation System. All reactions included a negative (non-template) and positive control of reference *E. coli* strains supplied by AHRI, Dokki, Giza, Egypt.

**TABLE 1:** Primer sequences and amplicon sizes of target genes.

Target gene	The function of the target gene	Primers sequences (5′-3′)	Amplicon size bp	References
*fim*H	Fimbriae binding virulence gene	TGCAGAACGGATAAGCCGTGG	508	Ghanbarpour & Salehi 2010
		GCAGTCACCTGCCCTCCGGTA		
*Stx*1	Shiga toxin 1 virulence gene	ACACTGGATGATCTCAGTGG	614	Dipineto et al. 2006
		CTGAATCCCCCTCCATTATG		
*Stx*2	Shiga toxin 2 virulence gene	CCATGACAACGGACAGCAGTT	779	Dipineto et al. 2006
		CCTGTCAACTGAGCAGCACTTTG		
*eae*A	Intimate attachment virulence gene	ATGCTTAGTGCTGGTTTAGG	248	Bisi-Johnson et al. 2011
		GCCTTCATCATTTCGCTTTC		
*hly*	Haemolysin virulence gene	AACAAGGATAAGCACTGTTCTGGCT	1177	Piva et al. 2003
		ACCATATAAGCGGTCATTCCCGTCA		
*omp*A	Outer membrane protein A virulence gene	AGCTATCGCGATTGCAGTG	919	Ewers et al. 2007
		GGTGTTGCCAGTAACCGG		
*tet*A	Tetracycline resistant gene	CCTTATCATGCCAGTCTTGC	576	Sabarinath et al. 2011
		ACTGCCGTTTTTTCGCC		
*bla*_CTX_-M	β-lactam resistant gene	ATGTGCAGYACCAGTAARGTKATGGC	593	Archambault et al. 2006
		TGGGTRAARTARGTSACCAGAAYCAGCGG		

*Source*: Please see the full reference list of the article for more information

bp, base pair.

### Gene sequencing of *fim*H gene

Two retrieved *E. coli* isolates one from diarrhoeic cattle and the other from Hooded crow were selected for genotyping of the *fim*H gene. QIAquick^®^ Gel Extraction Kit (QIAGEN, Germany) was used to purify the PCR products. The sequence reactions were performed using the BigDyeTM Terminator version 3.1 Cycle Sequencing Kit (Thermo Fisher Scientific, United States [US]) and then were analysed using the 3130 Genetic Analyzer (Applied Biosystems™, US). The obtained sequences were trimmed, consensus generated and analysed using the Uniport Ugene software version 43.0 (Okonechnikov et al. 2012). Sequences were investigated using the National Center for Biotechnology Information (NCBI) online basic local alignment tool (BLAST at https://blast.ncbi.nlm.nih.gov/Blast.cgi). For phylogenetic analysis, *fim*H gene sequences from different sources and origins including Egypt were selected and retrieved from the GenBank in FATSA format. Sequence alignments (MSA) were performed using MUSCLE algorism with the same software Multiple (Okonechnikov et al. 2012). IQ-TREE was used to construct the phylogenetic tree (Nguyen et al. 2014) using the maximum likelihood method, best model finder and 1000 bootstrap replicates for both nucleotide and protein sequences. The constructed trees were annotated using the Interactive tree of life (iTOL) (Letunic & Bork 2021).

## Results

### Phenotypic characterisation of the recovered *Escherichia coli* isolates in cattle and wild birds

The suspected *E. coli* colonies revealed a pink colouration on MacConkey agar medium, metallic green sheen on EMB and some (enterohaemorrhagic) strains gave haemolysis on blood agar. Microscopically, the isolates appear as Gram-negative rod-shaped motile bacilli. Also, their pathogenicity was confirmed on Congo red medium (gave positive orange or bright red colonies). Biochemically, they all were identified and confirmed as *E. coli* spp.

### The isolation rate of *Escherichia coli* in cattle and wild birds

Bacterial examination of 120 rectal swabs of both apparently healthy and diarrhoeic cattle revealed that *E. coli* was recovered from 55 of 120 (45.8%) animals. It was detected in 17 of 60 (28.3%) apparently healthy animals; however, it was isolated from 38 of 60 (63.3%) diarrhoeic animals. Moreover, in wild birds, 39 (32.5%) were positive for *E. coli,* of which 18 of 21 (85.7%) were isolated from the hooded crow, 10 of 18 (55.6%) from cattle egret, pied kingfisher 3 of 24 (12.5%), spur-winged plover 3 of 21 (14.3%) and 5 of 12 (41.7%) from stone curlew (Table 2).

**TABLE 2:** The prevalence of different *Escherichia coli* serotypes in cattle and wild birds.

Species	Source of samples	Number of positive *E. coli*			Serotypes of *E. coli* isolates							
		*N*	*n*	%	O26	O114	O128	O125	O78	O111	O55	O44
Cattle	Apparently healthy	60	17	28.3	2	4	2	3	1	3	2	-
	Diarrhoeic cattle	60	38	63.3	12	8	6	4	3	-	3	2
	Total cattle	120	55	45.8	14†	12†	8†	7†	4†	3†	5†	2†
Wild bird	Hooded crow	21	18	85.7	3	4	1	5	1	2	2	-
	Cattle egret	18	10	55.6	3	2	-	1	1	3	-	-
	Pied kingfisher	24	3	12.5	1	1	-	-	1	-	-	-
	Spur-winged plover	21	3	14.3	-	1	-	1	-	1	-	-
	Stone curlew	12	5	41.7	-	-	-	3	-	2	-	-
	Green bee-eater	24	0	0.0	-	-	-	-	-	-	-	-
	Total wild bird	120	39	32.5	7‡	8‡	1‡	10‡	3‡	8‡	2‡	0‡

**Total isolates**		**240**	**94**	**-**	**21** §	**20** §	**9** §	**17** §	**7** §	**11** §	**7** §	**2** §

† , Number out of 55;

‡ , Numer out of 39;

§ , Number out of 94.

### Serological identification of recovered isolates

Serological identification revealed different serotypes, according to O-antigen in which the most predominant serotype was O26 (*n* = 21/94, 22.4%), followed by O114 (*n* = 20/94, 21.3%), O128 (*n* = 9/94, 9.6%), O125 (*n* = 17/94, 18.1%), O111 (*n* = 11/94, 11.7%), O78 (*n* = 7/94, 7.4%), O55 (*n* = 7/94, 7.4%) and O44 (*n* = 2/94, 2.1%). Enterohaemorrhagic (EHEC) *E. coli* serovars were screened in this study in 38 of 94 (40.4%), 16 of 94 (17%) were enterotoxigenic (ETEC), 29 of 94 (30.9%) were EPEC and 11 of 94 (11.7%) were enteroaggregative (EAEC) as shown in Table 2.

### Antibiotic resistance pattern

Most examined *E. coli* isolates demonstrated high multidrug resistance levels against tetracycline and chloramphenicol (95.7% and 93.6%), respectively, followed by piperacillin, penicillin and streptomycin (90.4%, 88.3% and 88%), respectively. A moderate resistance level was recorded against both fosfomycin (54.3%) and amoxiclav (47.9%). Meanwhile, gentamycin, cefepime, ciprofloxacin and danofloxacin recorded the lowest resistance rates (9.6% and 10.6%, 12.8% and 26.6%), respectively, as shown in Table 3.

**TABLE 3:** Phenotypic characterisation of antibiotic resistance profile of *Escherichia coli* isolates.

Chemotherapeutic group	Chemotherapeutic agents/disc Potency μg/disc	No. of resistant strains		
		*N*	*n*	%
Aminoglycoside	Gentamycin (CN) (10 μg)	94	9	9.6
	Streptomycin (S) (10 μg)	94	78	83.0
Cephalosporins 4th generation	Cefepime (FEP) (30 μg)	94	10	10.6
Fluoroquinolones	Ciprofloxacin (CIP) (5 μg)	94	12	12.8
	Danofloxacin (DAN) (5 μg)	94	25	26.6
Other	Fosfomycin (FF) (50 μg)	94	51	54.3
Penicillins β-lactamase inhibitors	Piperacillin (PRL) (100 μg)	94	83	88.3
	Penicillin (P) (10 μg)	94	85	90.4
	Amoxiclav (AMC) (30 μg)	94	45	47.9
Chloramphenicol	Chloramphenicol (C) (30 μg)	94	88	93.6
Tetracycline	Tetracycline (T) (30 μg)	94	90	95.7

### Polymerase chain reaction investigations of virulence and resistant attributes of the recovered isolates

**FIGURE 1:**
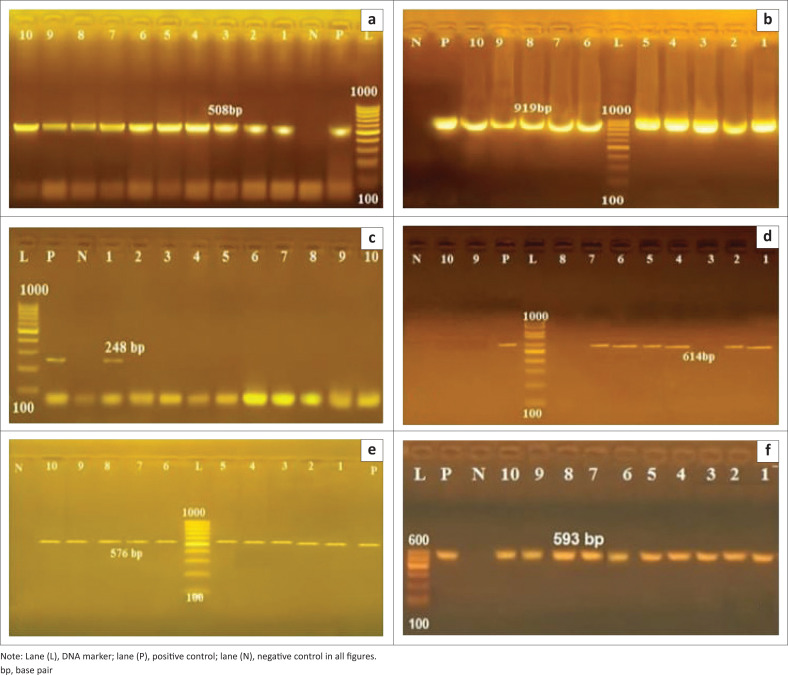
The electrophoretic gel pattern of MDR genes from *E. coli* isolates: (a): lane 1–10 positive for *fim*H at 508 bp, (b): lane 1–10 positive for *omp*A at 919 bp, (c): lane 1 positive for *eae*A at 248 bp, (d): lane 1, 2, 3, 4, 5, 6, 7 positive for *Stx*1 at 614 bp, (e): lane 1–10 positive for *tet*A at 576 bp, (f): lane 1–10 positive for *bla*_CTX_-M at 593 bp.

Polymerase chain reaction screening of the virulence genes of most 10 multi-drug resistant (MDR) *E. coli* isolates (Figure 1a–f) revealed that *fim*H and *omp*A genes were detected in all isolates 10 of 10 (100%) (Figure 1a and b), while the *eae*A gene was detected in 1 of 10 (10%) only of total isolates (Figure 1c), *Stx*1 gene was demonstrated in 6 of 10 (60%) of MDR isolates (Figure 1d), PCR failed to detect *hly* and *Stx2* genes in the examined *E. coli* isolates. The presence of *tet*A and *bla*_CTX_-M genes of tetracycline and penicillin β-lactamase inhibitors resistance was confirmed in all 10 of 10 (100%) of MDR tested *E. coli* isolates (Figure 1e and f) as data shown in Table 4.

**TABLE 4:** Polymerase chain reaction amplification results of different virulence and resistant genes of isolates.

Isolates no.	Source of isolates	Serotype	Antimicrobial susceptibility	Virulence genes						Resistant genes	
				*fim*H	*omp*A	*eae*A	*Stx*1	*Stx*2	*hly*	*tet*A	*bla*_CTX_-M
1	Hooded crow	O_125_ EHEC	CN,FEP,C,T,AMC,FF,PRL,S,CIP,DAN,P	+	+	+	+	-	-	+	+
2	Cattle egret	O_111_ EHEC	CN,FEP,C,T,AMC,FF,PRL,S,CIP,DAN,P	+	+	-	+	-	-	+	+
3	Stone curlew	O_125_ EHEC	CN,FEP,C,T,AMC,FF,PRL,S,CIP,DAN,P	+	+	-	-	-	-	+	+
4	Pied kingfisher	O_26_ STEC	CN,FEP,C,T,AMC,FF,PRL,S,CIP,DAN,P	+	+	-	+	-	-	+	+
5	Spur-winged plover	O_114_ STEC	CN,FEP,C,T,AMC,FF,PRL,S,CIP,DAN,P	+	+	-	+	-	-	+	+
6	Cattle	O_128_ STEC	CN,FEP,C,T,AMC,FF,PRL,S,CIP,DAN,P	+	+	-	+	-	-	+	+
7	Cattle	O_78_ STEC	CN,FEP,C,T,AMC,FF,PRL,S,CIP,DAN,P	+	+	-	+	-	-	+	+
8	Cattle	O_55_ EHEC	CN,FEP,C,T,AMC,FF,PRL,S,CIP,DAN,P	+	+	-	-	-	-	+	+
9	Cattle	O_44_ EHEC	CN,FEP,C,T,AMC,FF,PRL,S,CIP,DAN,P	+	+	-	-	-	-	+	+
10	Cattle	O_26_ EHEC	CN,FEP,C,T,AMC,FF,PRL,S,CIP,DAN,P	+	+	-	-	-	-	+	+

**Total (%)**				**100.0**	**100.0**	**10.0**	**60.0**	**0.0**	**0.0**	**100.0**	**100.0**

STEC, Shiga toxigenic *Escherichia coli*.

### Phylogenetic analysis of *fim*H virulence gene

**FIGURE 2:**
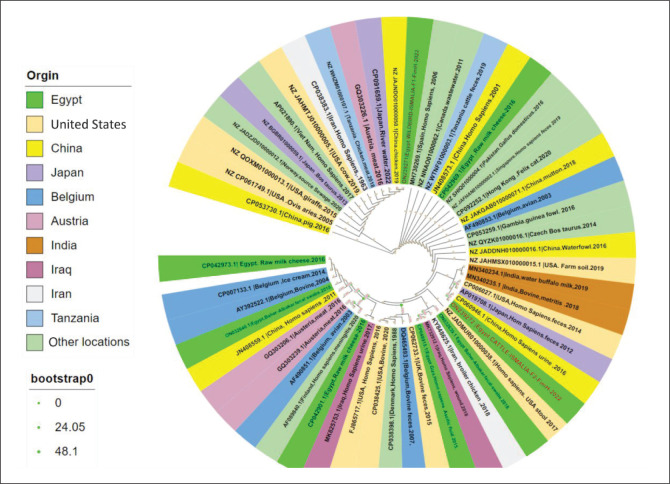
The maximum likelihood (ML) phylogenetic tree for partially nucleotide sequenced *Fim*H gene of *E. coli* isolated from wild bird and cattle. The tree was constructed in IQ-TREE with Model Finders. The accession number and a brief GenBank ID are assigned to each retrieved sequence from different l isolation sources and origins. The two isolates are highlighted in red with a green background, various clades are designated with different colours, and the numbers above the branches are the branch length. The bootstrap values were computed from 1000 bootstrap repeats and branch length as visualised by iTOL. The tree’s roots are in the middle.

**FIGURE 3:**
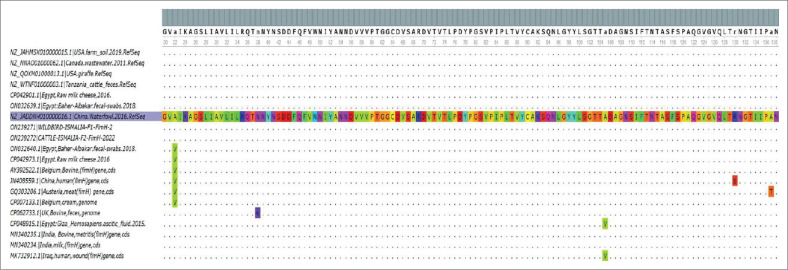
The MSA for partial nucleotide *fim*H gene fragment of the identified *Escherichia coli* isolates compared with other isolates and strains retrieved from the Gene Bank visualised by UGEN programme. The dot (.) represents identity, while a single alphabet highlights the differences among aligned sequences.

**FIGURE 4:**
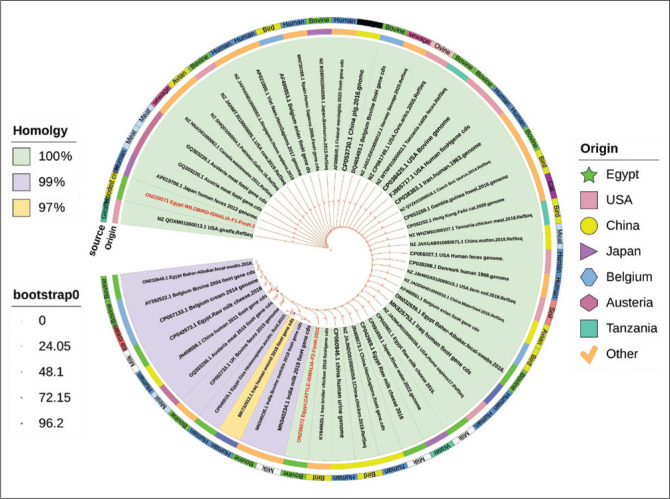
Rooted maximum likelihood phylogenetic tree for a translated protein of *fim*H gene *Escherichia coli* sequences obtained from wild bird and cattle isolates. The branch colour represents the homology between protein sequences. Rings from inner to outer: the first ring (origin) represents the geographical location of selected isolates, the second ring (source) is for the sample source of isolates, the branch length, and bootstrap values computed from 1000 bootstrap repeats are also visualised.

The *fim*H was detected in all examined isolates with the conventional PCR technique. *fim*H gene sequences from two selected wild birds (hooded crow) and cattle isolates were examined and then documented in the Gene Bank database and assigned the accession numbers (ON239271 and ON239272), respectively. The phylogenetic analysis of the *fim*H gene selected from two isolates revealed 99% nucleotides identity between their sequences (cattle and wild bird isolates, Figure 2). Only three single nucleotides polymorphism (SNP) in cattle isolates were shown at positions 192.291,348, but these SNPs did not show any effect at amino acid (a.a) level because they were identical (100% identity, Figure 3). Both cattle and wild bird isolates showed 100% homology with most sequences retrieved from the GenBank from Egypt and geographical locations also from different isolation sources including different animal species, food and environmental source (Table 5). These translated a.a phylogeny results confirmed the high conservation level of the sequenced *fim*H gene for both retrieved Egyptian *E. coli* isolates and globally (Figure 4).

**TABLE 5:** Source modifier tabulates for *fim*H gene isolates and strains sequences retrieved from GenBank for alignment, phylogenetic analysis, and tree construction for isolates from wilds birds and cattle in Egypt.

Country (location)	Sequence identification	GenBank accession number	Collection date	Host and sources
Egypt	E. coli-WILD, BIRD-ISMALIA-F1-*fim*H-2022	ON239271	2022	Wild bird (Hooded crow)
Egypt	E. coli-CATTLE-ISMALIA-F2-*fim*H-2022	ON239272	2022	Cattle (diarrhoea)
Egypt	Strain CFSAN061769 chromosome, complete genome	CP042969.1	2016	Raw milk cheese
Egypt	strain CFSAN061762 chromosome, complete genome	CP042901.1	2016	Raw milk cheese
Egypt	strain GH_12 *fim*H (*fim*H) gene, partial CDs	ON032639.1	2018	Cow (faeces)
Egypt	strain E2 chromosome, complete genome	CP048915.1	2015	*Homo sapiens* (Ascitic fluid)
Egypt	strain CFSAN061768 chromosome, complete genome	CP042973.1	2016	Raw milk cheese
Egypt	strain GH_13 *fim*H (*fim*H) gene, partial CDs	ON032640.1	2018	Cow (faeces)
China	TMUSHC1839 *fim*H (*fim*H) gene	JN408573	2011	*Homo sapiens*
The Gambia	strain GF3-3 chromosome, complete genome	CP053259.1	2016	Guinea fowl
Iran	O55:H7 strain DEC5E chromosome, complete genome	CP038383.1	1963	Homo sapiens
Hong Kong	strain ISHS7 chromosome, complete genome	CP092252.1	2020	Felix cat (faeces)
Belgium	isolate APEC 17 *fim*H (*fim*H) gene, complete cds	AF490853.1	2003	Avian
Vietnam	2017.15.01CC DNA, complete genome	AP021890.1	2017	*Homo sapiens*
Japan	strain KFu024 chromosome, complete genome	CP091659.1	2022	River water
Austria	strain 9m *fim*H (*fim*H) gene, partial cds	GQ303226.1	2016	Meat
China	strain CP61_Sichuan chromosome, complete genome	CP053730.1	2016	Pig (slaughterhouse)
China	strain TMUSHC1839 *fim*H (*fim*H) gene, partial cds	JN408573.1	2011	*Homo sapiens*
India	strain 97/K *fim*H adhesin (*fim*H) gene	MN340234.1	2019	Bubalus bubalis (milk)
India	strain NZ/3/G-10/12 *fim*H adhesin (*fim*H) gene	MN340235.1	2018	Bos indicus (metritis)
United States	O145:H28 str. RM13514, complete genome	CP006027.1	2014	*Homo sapiens* (faeces)
Japan	O145:H28 122715 DNA, complete genome	AP019708.1	2012	*Homo sapiens* (faeces)
China	O157:H7 str. EC10 chromosome	CP060946.1	2016	*Homo sapiens* (urine)
United Kingdom	O157:H7 strain Z1767 chromosome	CP062733.1	2015	Bovine (faeces)
Belgium	strain EH485 adhesin (*fim*H) gene	DQ465493.1	2007	Bovine (faeces)
Denmark	O157:H7 strain DEC4E chromosome	CP038398.1	1988	*Homo sapiens*
United States	O157:H7 strain 2571 chromosome	CP038425.1	2020	Bovine
United States	strain TB2755 type 1 fimbrial adhesin (*fim*H) gene	FJ865717.1	2016	*Homo sapiens*
Iraq	strain ECO/irq.Hill12 *fim*H protein (*fim*H) gene, partial cds	MK732912.1	2018	*Homo sapiens* (wound)
Iran	fimbrial adhesin protein (*fim*H) gene	KY848625.1	2018	Broiler chicken (faeces)
Iraq	strain E17-R D-mannose binding protein (*fim*H) gene	MK825753.1	2017	*Homo sapiens* (urine)
United States	O157:H7 strain 2571 chromosome, complete genome	CP038425.1	2020	Bovine
Finland	Escherichia coli *fim*H (*fim*H) gene	AF089840	2020	*Homo sapiens* (meningitis)
Belgium	isolate APEC 15 *fim*H (*fim*H) gene	AF490851.1	2003	Avian
Austria	strain 90mb *fim*H (*fim*H) gene	GQ303239.1	2016	Meat
Austria	strain 76s *fim*H (*fim*H) gene	GQ303206.1	2016	Meat
China	strain TMUSHB3626 *fim*H (*fim*H) gene	JN408559.1	2011	*Homo sapiens*
Belgium	O145:H28 str. RM12761,	CP007133.1	2014	Ice cream
Belgium	clone B1290 *fim*H (*fim*H) gene	AY392522.1	2004	Bovine clinical case
United States	strain BSD2780120874b_170522_F6 NODE_38_length_41060_cov_32.1749	NZ_JADMUR010000038.1	2017	*Homo sapiens* (stool)
China	strain 18FS24-1 NODE_16_length_111283_cov_38.446139	NZ_JADDNH010000016.1	2016	Waterfowl
Singapore	strain 2EC2 contig_2, whole genome shotgun sequence	NZ_JAFHAN010000002.1.	2019	*Homo sapiens* (faeces)
United States	strain KCJ2K2235 NODE_5_length_345482_cov_18.390569	NZ_JAHMTJ010000005.1.	2019	Cow
China	strain w183 NODE_50_length_27035_cov_162.619	NZ_JAJNDO010000050.1.	2019	Chicken
Pakistan	strain EC_43 NODE_4_length_262439_cov_19.018654	NZ_SHIQ01000004.1	2016	Gallus domesticus
Canada	strain WW223 NODE_62_length_30559_cov_31.906	NZ_NNAO01000062.1	2011	Wastewater
United States	strain KCJ2K2539 NODE_15_length_112297_cov_10.368824	NZ_JAHMSX010000015.1	2019	Farm soil
Tanzania	strain 10432wG5 NODE_3_length_591091_cov_50.1463	NZ_WTNF01000003.1	2018	Cattle faeces
Tanzania	strain 10432wG5 NODE_3_length_591091_cov_50.1463	NZ_WHZM01000107.1	2018	Chicken meat
Czech	strain ET33 contig016_Escherichia_coli	NZ_QYZK01000016.1	2014	Bos taurus
China	strain STEC814 Contig_71_174.05,	NZ_JAKGAB010000071.	2018	Mutton
United States	O15:H12 strain 2273-PO3 chromosome	NZ_CP061749.1	2025	Ovis aries
United States	strain MOD1-ECOR27 ECOR27_13_length_156310_cov_130.462	NZ_QOXM01000013.1	2015	Giraffe
Japan	KS-P089 DNA, sequence59	NZ_BGBR01000059.1	2013	Bos taurus
Norway	strain 2-346 NODE_12_length_116060_cov_25.614484	NZ_JADZJD010000012.1	2020	Sewage

STEC, Shiga toxigenic *E. coli*; DNA, deoxyribonucleic acid.

## Discussion

Many studies implicated the crucial role of wild birds in the pathogenesis of *E. coli* spp. in livestock animals (Fadel, Afifi & Al-Qabili 2017; Fahim et al. 2019). However, its pathogenesis in these birds was still unclear. Diarrhoeagenic strains of *E. coli* were isolated from different wild bird species including migratory and non-migratory (Ahmed et al. 2019), Passeriformes, Columbiformes and Pelecaniformes (Sanches et al. 2017). *Escherichia coli* also was isolated from birds of prey, waterfowls and passerines. Farm animals could infect wild birds or vice versa (Fadel et al. 2017). They infiltrated animal enclosures in search of water and food hence infecting them with different pathogens or even acquiring the infection from these animals. Moreover, the feeding practice of cattle in open yards could result in the accumulation of their manure and so the attraction of these wild birds to those farms (Medhanie et al. 2015).

The present article stated that *E. coli* was detected higher (45.8%) from all examined apparently healthy and diarrhoeic cattle than from different wild birds (32.5%). For apparently healthy cattle, it was detected in 17 of 60 (28.3%); however, it was isolated from 38 of 60 (63.3%) of diarrhoeic animals. However, from different wild bird species, 18 of 21 isolates were obtained from the hooded crow (85.7%), 9 of 18 (50%) cattle egret, pied kingfisher (*n* = 3/24, 12.5%), spur-winged plover (*n* = 3/21, 14.3%), 6 of 12 (50%) from stone curlew.

In the same way, recent studies discussed the propagation rate of *E. coli* in wild birds, cattle and their environment in which it was found in a range of 17% – 47% in the faeces samples of wild birds (house sparrows, red-winged blackbirds, European starling and brown-headed cowbirds) despite its percentage was recorded higher in cattle farms (Tormoehlen et al. 2019). Also, 478 positive *E. coli* samples of migratory birds were reported in China from a total of 1387 (34.7%) faecal, cloacal and throat samples (Yuan et al. 2021). Ahmed et al. (2019) isolated *E. coli* from 60% and 45% of examined hooded crows and cattle egrets in Egypt, respectively. However, a higher rate of *E. coli* was recovered from the faeces of wild birds (70%) than migratory waterfowls (33.3%) (Fahim et al. 2019). From a different point of view, a large number of *E. coli* were isolated from egret wild birds than from cattle in the same study (Fashae et al. 2021) This variation in the isolation rate in different studies might be because of either insensitivity testing method or other anonymous agents (Ballem et al. 2021).

Moreover, *E. coli* isolates were recorded also in the United States (21% of beef cattle and 13% of dairy cattle) (Venegas-Vargas et al. 2016). Also, 112 of 409 positive *E. coli* isolates were retrieved from cattle in Portugal with a prevalence of 27.4%, and 133 STEC isolates were identified (Ballem et al. 2021). In addition, 106 and 29 *E. coli* isolates were yielded from 77 diarrheic and in-contact calves (Awad et al. 2020).

From a serological view, the most predominant type of *E. coli* in this study was O26 (*n* = 26/94), followed by O114, O128, O125, O111, O78, O55 and O44 (Table 2). Corresponding results were reported by (Mahmoud et al. 2015; Navarro-Gonzalez et al. 2020) in which different subtypes of *E. coli* O26, O55, O111, O124, O119, O114, O26, O44 and O163 were recorded.

Antibiotic-resistant bacteria could pose a rising hazard to global public health and accompanied environmental contamination problems (WHO 2017). The Regulation (EC) no. 1831/2003 of the European Parliament and of the council of 22 September 2003 on additives for use in animal nutrition banned the use of growth-promoting antimicrobials in animal production. As a result of the diversity in ecological niches, the migratory birds act as reservoirs and transporters of antibiotic-resistant bacteria and consequently play a significant epidemiological role in the dissemination of antibiotic-resistant genes (ARGs) (Cao et al. 2020). These birds could carry ARGs during migration leading to the dissemination of MDR bacteria and ARGs through the environment (Yuan et al. 2021).

The presented information in this study displayed MDR phenomena of the yielded isolates because they showed high antimicrobial resistances against tetracycline and chloramphenicol with prevalence rates of 95.7% and 93.6%, respectively, followed by piperacillin, penicillin and streptomycin (90.4%, 88.3% and 88%), respectively. Meanwhile, gentamycin, cefepime, ciprofloxacin and danofloxacin were highly sensitive where the lowest resistance was recorded (9.6% and 10.6%, 12.8% and 26.6%), respectively, as shown in Table 3. These results might be of good importance in management routines for cattle farms to control the spread of antimicrobial resistance.

Similar to this, the wild bird *E. coli* isolates exhibited bacterial resistance in many studies in the last years. *Escherichia coli* isolates showed great resistance to penicillin G, piperacillin, tetracycline, cotrimoxazole, ampicillin and nitrofurantoin (Shinde et al. 2020). Also, 376 recovered *E. coli* isolates from Hooded and White-naped cranes in Japan were found resistant to oxytetracycline, ampicillin and nalidixic acid antibiotics. A high resistance level was also recorded against tetracycline followed by sulfamethoxazole, ampicillin, trimethoprim and ciprofloxacin in most *E. coli* isolates (Suenaga et al. 2019). Furthermore, 87 of 88 egret’s and 53 of 55 cattle *E. coli* isolates were found to have MDR against more than one antimicrobial. Tetracycline resistance was highest in *E. coli* isolates from egret birds (*n* = 85/87), further followed by streptomycin (*n* = 69/87) and ciprofloxacin resistance (*n* = 38/87).

For cattle *E. coli* isolates multiple authors reported MDR patterns of *E. coli* isolates (Iweriebor et al. 2015, Mahmoud et al. 2020). The MDR phenomena are of great concern because the resistant strain could be transmitted to humans by consumption of either milk or food carrying antibiotic-resistant bacteria, which could lead to the acquisition of antibiotic-resistant infections (Geletu, Usmael & Ibrahim 2022), and also could be transmitted to accompanying animals and their offspring (Roca-Saavedra et al. 2018). It was previously documented that resistant strains selected during an antimicrobial treatment last for a long time in the intestinal tract when this treatment ceases. In addition, these resistant strains could modify animal health.

The results in this study were consistent with Geletu et al. (2022) who revealed that tetracycline (80%) was the drug that most *E. coli* isolates from dairy cattle were extremely resistant to, followed by ceftriaxone and vancomycin (83%). However, gentamycin (90%) and nitrofurantoin (70%) were the most sensitive drugs, respectively. Additionally, tetracycline resistance (*n* = 47/53) was the most often seen phenotype in cow cefotaxime-resistant *E. coli*, followed by streptomycin (*n* = 46/53) and ciprofloxacin (*n* = 17/53) resistance (Fashae et al. 2021). Moreover, Mahmoud et al. (2015) recorded the *E. coli*. resistance against oxytetracycline and ampicillin in cattle samples. Moreover, the most responsive medications, nevertheless, were ceftiofur (40%) and lincospectine (56.6%), followed by danofloxacin (56.6%), enrofloxacin (40%) and danofloxacine (56.6%). Furthermore, sulfamethoxazole, ampicillin, trimethoprim and ciprofloxacin were the antibiotics with the highest rates of resistance in *E. coli* isolates, followed by tetracycline (Hang et al. 2019). In the present study, the highest resistance levels of *E. coli* isolates might be because of the non-judicious use of antibiotics on a cattle farm. Also, this high antimicrobial resistance of *E. coli* isolates in cattle might confer a selective advantage towards intestinal colonisation, which might itself increase the faecal shedding of antimicrobial-resistant *E. coli* (Harkins, McAllister & Reynolds 2020).

Studying the genotypic virulence attributes of the isolated bacterial species was applied by conventional PCR technique. Our results indicated that the *fimH* gene, one of the virulence genes involved in bacterial adhesion, was found to be present in 10 of 10 (100%) of the tested *E. coli*. Also, identical findings were recorded by Nüesch-Inderbinen (et al. 2018) who discovered that the *fimH* gene was present in all (100%) of their isolates. In the present study, *eae*A (attaching and effacing virulence factor) was detected in 1 of 10 (10%) of tested *E. coli* isolates, despite this finding complied with a study by Sanches et al. (2017) in which *eae*A gene was found in a rate of 5.74%. Moreover, Mohamed and Sayed (2017) implied that *eae*A gene was exhibited in 43.75% of yielded *E. coli* isolates. These results disagreed with the finding by Nüesch-Inderbinen et al. (2018) who recorded that *eae*A gene was not present in any of the studied *E. coli* isolates.

Depending on the retrieved data in this study, PCR confirmed the positivity of the *omp*A gene (outer membrane protein A) virulence gene in 10 of 10 (100%) of *E. coli* isolates. The frequency of detected *omp*A gene in our study was much higher than another study in which this gene was detected in (82%) of *E. coli* isolates (Ammar et al. 2015).

While the presence of virulence factors such as Shiga toxin (*Stx1* and *Stx2*) and α-haemolysin (*hly*) of *E. coli* is pivotal for suggesting the increased pathogenicity of these strains, serogroups are still crucial for identifying potential diseases. In this study, the prevalence of *Stx*1 virulence gene was 6 of 10 (60%), while (*Stx*2 and *hly*) genes failed to be detected. In accordance with the recorded result, Nasef, El Oksh and Ibrahim (2017) detected *Stx*1, *Stx*2 and *hly* (45%, 65% and 80%), respectively.

Studying the antimicrobial genotypic attributes of the isolates was applied by PCR to investigate the presence of tetracycline and penicillin β-lactams resistance gene (*tet*A and *bla*_CTX_-M), the results indicated positive detection in all 10 of 10 (100% for each) of the tested *E. coli*. Similar outcomes were reported by (Fashae et al. 2021) who detected *the bla*_CTX_-M gene in 83.3% of MDR *E. coli* isolates. Furthermore, according to Gholami-Ahangaran et al. (2021), all *E. coli* isolates from faecal samples of pet birds included the *tet*A gene.

The phylogenetic analysis of the *fim*H gene of two selected *E. coli* isolates from both resident free-living wild birds and cattle, which was in contact, demonstrated a high conservation level of the gene at (a.a) level as previously proved by Vandemaele, Hensen and Goddeeris (2004). The homology was 100% with gene sequence from different resources including avian, cattle, birds, pig and from food sources such as milk and ice cream and also from environmental sources such as water, sewage and farm soil prove the potential role of wild birds as a reservoir for *E. coli* having MDR genes. This was concordant with results mentioned by Nabil et al. (2020). However, the detected SNPs between both examined samples represented no change at protein level proves the common source nature of pathogens suggesting the possible role of wild birds to contaminate water sources and disseminating the infection to cattle farms (Fahim et al. 2019). Also, our findings could highlight the public health concern of presence of wild birds carrying *E. coli* with MDR genes in contact with dairy cattle farms and its surrounding environment that could transmit infection to human through the food chain.

## Conclusion

This study reported updated data about the critical infectious role of faecal matter of wild birds to cattle farm. Highly virulent and resistant pathogenic serovar of *E. coli* could be disseminated towards different animal species triggering several diseases, threatening their health and impairing the animal farm economy. Hence, strict recommendations for animal manure with good hygienic practices in cattle farms should be implicated. Also, one health approach should be implicated to inherit the dispersion of multiple antimicrobial resistance phenomena. Furthermore, more advanced sequencing approaches should be studied on the whole genome level for such bacteria to indicate the interrelationship of virulent and resistant genes in different animal, human and wild bird species.
